# Eurycoma longifolia (Tongkat Ali) supplementation enhances sleep and wake consolidation in wild-type, but not in narcoleptic mice

**DOI:** 10.1093/sleepadvances/zpae047

**Published:** 2024-07-10

**Authors:** Noriaki Sakai, Kazuhiro Komi, Naoya Nishino, Yutaka Kuroki, Seiji Nishino

**Affiliations:** Department of Psychiatry and Behavioral Sciences, Stanford University School of Medicine, Palo Alto, CA, USA; Center for Doctors’ Career Development, Kawasaki Medical School Hospital, Kurashiki, Japan; Department of Psychiatry and Behavioral Sciences, Stanford University School of Medicine, Palo Alto, CA, USA; D-LAB, Japan Tobacco Inc, Tokyo, Japan; Delightex Pte. Ltd., Bugis Junction Towers, Singapore; Department of Psychiatry and Behavioral Sciences, Stanford University School of Medicine, Palo Alto, CA, USA

**Keywords:** tongkat ali, sleep–wake consolidation, narcolepsy, blood hormone

## Abstract

Tongkat Ali (TA), also known as Eurycoma longifolia, has been used as a traditional herbal medicine for anti-aging, evidenced by clinical trials presenting the beneficial effects on energy, fatigue, and mood disturbance. We have recently shown that TA supplementation dose-dependently enhances the rest–activity pattern in C57BL/6 mice. Since destabilization of wakefulness and sleep is one of the typical symptoms of not only the elderly but also narcolepsy, we performed sleep analysis with and without dietary TA extract supplementation in middle-aged (10–12 months old) wild-type (WT) and narcoleptic DTA mice. We found that TA supplementation enhanced diurnal rhythms of locomotion and temperature in a time-of-day-dependent manner in WT mice but attenuated in DTA mice. In WT mice, TA supplementation consolidated wakefulness with a long bout duration and led to less entries into the sleep state during the active period, while it consolidated NREM sleep with long bout duration during the resting period. Neither disturbed sleep and wake cycles nor cataplexy was sufficiently improved in DTA mice. EEG spectral analysis revealed that TA supplementation enhanced slow wave activity (SWA) at both delta and low delta frequencies (0.5–4.0 and 0.5–2.0 Hz) during the light period, suggesting TA extract may induce vigilance during the active period, which then elicits a rebound effect during the resting period. Interestingly, DTA mice also slightly, but significantly, increased SWA at low frequencies during the light period. Taken together, our results suggest that TA supplementation enhances the Yin-Yang balance of sleep, temperature, and locomotion in WT mice, while its efficacy is limited in narcoleptic mice.

**Statement of Significance**

Clinical and preclinical studies suggest that Tongkat Ali extract improves vigor and vitality. For this reason, the extract has been used as a traditional herbal medicine for anti-aging in Southeast Asia. Our previous study reported that the extract supplementation dose-dependently enhanced the rest–activity pattern in middle-aged C57BL/6 mice. It is therefore informative to assess the therapeutic potential to manage excessive daytime sleepiness in narcoleptic mice. This study helps to better understand the usefulness of natural products and accelerates research to fill important unmet clinical needs in hypersomnia.

Tongkat Ali (TA), also known as Eurycoma longifolia, is a native plant found in Southeast Asia, and the roots commonly called Malaysian Ginseng have been used as a traditional herbal medicine for anti-aging benefits such as resilience to stress and anxiety [1, 2], enhancement of muscle strength [3], and aphrodisiac effects [4]. A clinical trial subjected to middle-aged men showed that TA extract supplementation improved the total testosterone levels, aging male symptoms, and fatigue [5]. Another trial also demonstrated that daily supplementation with TA extract improved stress hormone profiles and psychological mood state parameters, including tension, anger, and confusion, in moderately stressed male and female participants [2]. In addition, a recent randomized controlled trial indicated that TA supplements had the potential to improve stress markers and consequently improve sleep quality in healthy adults [6]. While the mechanisms of action of TA remain largely unknown, its beneficial effects on energy, fatigue, and mood disturbance may contribute to a better Yin-Yang balance between sleep and wakefulness, and vice versa.

Narcolepsy is a chronic neurological disorder characterized by excessive daytime sleepiness, cataplexy, disrupted nighttime sleep, hallucinations, and sleep paralysis [7, 8]. In addition to the abnormalities in vigilance and maintenance of wakefulness, narcoleptic patients commonly experience depressed mood and severe fatigue, resulting in severe functional impairment and low quality of life [9, 10]. Based on many advances in our understanding of the neuropathology of narcolepsy, development of new treatments including selective hypocretin/orexin receptor agonists are underway [11]. However, current treatments for narcolepsy are still symptomatic and each medication is associated with limitations, such as issues of tolerability, abuse potential, and suboptimal response.

Our group and others recently reported that feeding B6 mice with TA extract supplementation dose-dependently amplified the rest–activity pattern compared to a control diet: mice were more active during the dark period and more restful during the light period [12, 13]. Notably, TA enhanced wakefulness during the second half of the active period. As sleep propensity tends to accumulate towards the end of the active period, TA may have the potential as a wake-enhancing agent for daytime sleepiness. Destabilization of wakefulness and sleep is a well-described symptom of not only the elderly but also narcolepsy [14–16]. Although the mechanism underlying the time-of-day-dependent effects in mice is still unknown, it is of great interest to examine whether TA extract can improve hallmark symptoms of narcolepsy, such as disrupted sleep–wake cycles and cataplexy. In this study, we performed sleep analysis with and without TA extract supplementation in both wild-type (WT) and narcoleptic DTA mice, comparing the responses in each genotype. To explore the mechanism of action, we analyzed blood hormone levels (corticosterone/testosterone) during light and dark periods in C57BL/6 mice. The activation of hypocretin neurons involved in wakefulness was also examined by immunohistochemistry.

## Materials and Methods

### Materials

A regular rodent diet (AIN-93M, #D10012M, Research Diets, New Brunswick, NJ) was purchased as a control diet. TA extract powder (Phytes Biotec Sdn Bhd, Selangor, Malaysia), provided by Delightex. Pte. Ltd (Singapore), was incorporated into the AIN-93M at a concentration of 0.5% by mass, The mixture was pelleted by Research Diets, Inc. to create the test compound diet. All materials were stored at room temperature.

### Animals

Orexin-tTA mice were bred with B6.Cg-Tg(TetO-DTA)1Gfi/J mice (#8468, The Jackson Laboratory, Sacramento, CA) to produce *orexin-tTA; TetO DTA* mice that were hemizygous for both transgenes [17]. WT mice with no transgenes were used as controls. From the day of mating, all breeders and pups were given doxycycline-containing (DOX) chow (200 μg DOX/g chow, TD.98186, Envigo, Madison, Wisconsin), to deliver a daily dose of approximately 0.6 mg DOX. After weaning, WT and *orexin-tTA; TetO DTA* mice were fed with DOX chow until they were switched to standard chow (#2918, Envigo) at 7 weeks old. C57BL/6 mice aged 7–9 months were used for ELISA and immunohistochemistry. Males were used for all studies to exclude the influence of hormonal fluctuations over the estrous cycles. Animals were individually housed on a 12-hour light–dark cycle at room temperature at 23 ± 1°C. Food and water were available ad libitum.

All experimental procedures were approved by the Committee on the Ethics of Animal Experiments of the Stanford University Administrative Panel on Laboratory Animal Care and were conducted in accordance with the Stanford University Administrative Panel on Laboratory Animal Care Guidelines (APLAC-#21646). All efforts were made to minimize animal suffering or discomfort and to reduce the number of animals used.

### Telemetry implantation and EEG/EMG surgery

Eight males per genotype, aged 10–12 months, were anesthetized with 3% isoflurane and implanted with telemetry and EEG/EMG electrodes for sleep recording. A telemetry device (G2 E-Mitter; Mini Mitter OR, USA) was implanted in the abdominal cavity under 3% isoflurane anesthesia to measure locomotor activity and core body temperature. A headstage with 4 EEG and 2 EMG electrodes were surgically implanted on the skull where two EEG electrodes were screwed over the motor cortex (1.0 mm lateral and 1.0 mm anterior to bregma) and the other two electrodes over the visual cortex (3 mm lateral and 1 mm anterior to lambda). Two Teflon-coated stainless-steel wires for EMG were placed into the neck extensor muscles. Mice were singly housed and given a 2-week recovery period following surgery. During this time, their pellet diet was switched to the AIN-93M control diet.

### Sleep recording and analyses

**Figure 1.**
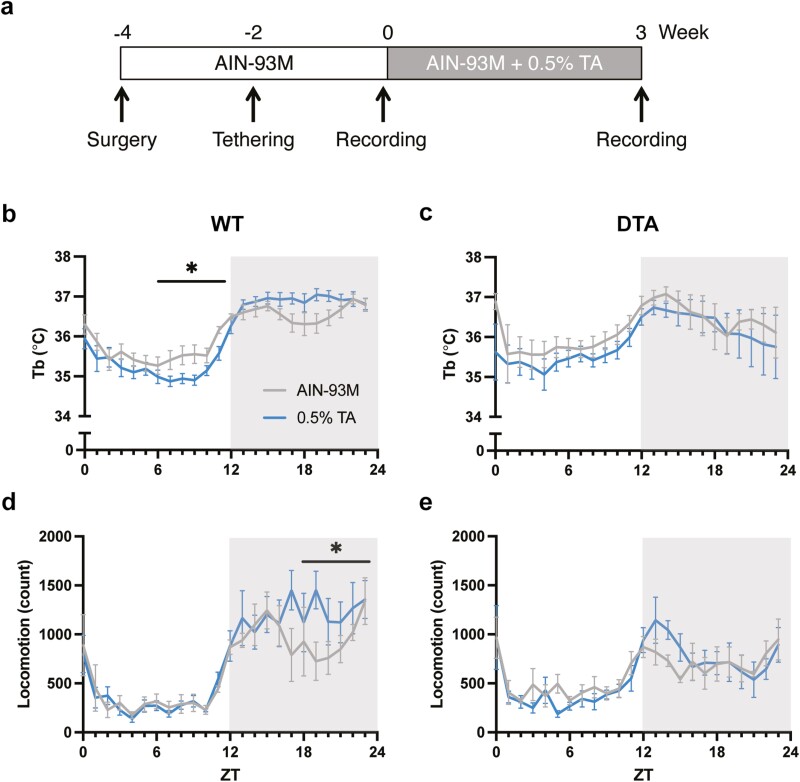
Core body temperature and locomotor activity rhythms in a 24-hour period. (A) A schematic diagram of the experimental design is shown. Time course of core body temperature (upper) and locomotor activity (lower) across 24 hours in (B, D) WT and (C, E) DTA mice. The gray and blue lines represent the group fed with the control AIN-93M diet or 0.5% TA-supplemented diet, respectively. All data are presented as mean ± SEM (*n* = 8 per group). Data were compared between the treatments over 24 hours (in 6-hour bins) by two-way repeated measures ANOVA. *p < .05

Mice implanted with telemetry and electrodes were tethered to recording cables that connect to a low-torque slip-ring commutator (Biella Engineering, Irvine, CA, USA). After 1 week of habituation to the cable and recording environment, consecutive recordings of EEG/EMG, locomotor activity, and core body temperature were started. Following the baseline recording day, the control diet was removed, and mice were maintained with a 0.5% TA diet for 3 weeks (Figure 1A). EEG and EMG signals were acquired using a Grass amplifier (West Warwick, RI, USA), filtered (30 Hz Low Pass Filter for EEG; 10–100 Hz Band Pass Filter for EMG), and captured at a sampling rate of 128 Hz using a sleep recording system (Vital Recorder; Kissei Comtec Co. Ltd., Matsumoto, Japan). Locomotor activity and core body temperature were monitored through VitalView software (Mini Mitter, OR, USA) and analyzed in 1-hour bins.

EEG and EMG signals were automatically scored in 10-second epochs using SleepSign software (Kissei Comtec Co. Ltd., Matsumoto, Japan). Following standard criteria, each epoch was classified as wake, NREM, or REM sleep. The automatic scoring output was visually examined and confirmed by an experienced scorer, blind to genotype and treatment. Cataplexy was visually scored according to the consensus definition of cataplexy [18–20]. Fast Fourier transform (FFT) was performed to analyze power spectra profiles of EEG over 0–30 Hz with 0.5 Hz bins for the δ (0.5–4 Hz) and θ (4–9 Hz) bandwidths in a state-specific manner. A relative EEG power spectrum was then calculated from EEG power densities in each frequency bin and normalized by the total bandwidth over 0–30 Hz. Epochs with artifacts and noise were excluded from the calculation.

### Blood collection and serum testosterone and corticosterone analysis

After 3 weeks of feeding, whole blood was collected via cardiac puncture from mice sacrificed at ZT 6 or ZT 18, incubating at room temperature for 30 minutes and centrifuged at 2000 × *g* for 15 minutes at 4°C. Serum hormone was measured using testosterone ELISA kit (NC1017824, Cayman) and corticosterone ELISA kit (ADI-900-097, Enzo Life Sciences) according to the manufacturer’s instructions.

### Immunohistochemistry

After blood collection, the brain was removed, and the hypothalamic area was fixed in 4% paraformaldehyde overnight at 4°C. Subsequently, brains were incubated in 30% sucrose solution at 4°C for 2 days. Brains were frozen in O.C.T. compound (Sakura Finetek) and cryosectioned with a CM1850 cryostat (Leica) at 35 µm thickness. Four series of coronal sections were stored in PBS with 0.02% sodium azide at 4°C. For hypocretin immunostaining, slide-mounted sections were permeabilized with 0.3% Triton X-100 in PBS for 30 minutes and blocked with 5% normal donkey serum (Jackson ImmunoResearch), 0.1% Tween-20 in PBS for 1 hour. Sections were incubated with antibodies against hypocretin (1:500, sc-8070, Santa Cruz) and c-fos (1:5000, 2250, Cell Signaling) overnight. After washing in PBS, sections were subsequently incubated with secondary antibodies conjugated with Alexa Fluor 488 (anti-goat, 1:500, Abcam) or Alexa Fluor 555 (anti-rabbit, 1:500, Abcam) for 1 hour. After washing in PBS, slides were mounted with Fluoroshield (Abcam). Images were captured using ZEISS Axio Imager.A2.

### Statistical analyses

Statistical analysis was performed using GraphPad Prism 9 (GraphPad Software Inc.). The sleep–wake parameters, time course changes in locomotor activity, core body temperature, and FFT power spectrum were analyzed using two-way repeated measures ANOVA (compound, time, frequency, and interaction). For multiple comparisons, Sidak’s multiple comparisons test was used when compared to the relevant control treatment. Paired *t*-tests were performed for each pair of state transitions. The hormone concentration and cell counts were analyzed using the Mann–Whitney test. The level of statistical significance was set at *p* < .05. All data are shown as mean ± SEM.

## Results

### TA-enhanced diurnal rhythms of temperature and locomotor activity were found in WT but were attenuated in narcoleptic mice

#### Core body temperature.

We reproduced the previous finding that TA supplementation altered core body temperature in WT mice in a time-of-day-dependent manner: TA extract decreased temperature in the light (resting) period and increased in the dark (active) period (control vs 0.5% TA at ZT0-5: 35.7 ± 0.2°C vs 35.4 ± 0.2°C, *p* = .70; ZT6-11: 35.6 ± 0.2°C vs 35.1 ± 0.1°C, *p* = .03; ZT12-17: 36.6 ± 0.1°C vs 36.8 ± 0.1°C, *p* = .70; ZT18-23: 36.6 ± 0.2°C vs 36.9 ± 0.1°C, *p* = .07; Figure 1B) [12, 13]. The distinct effect of TA extract during resting and active periods enhanced the diurnal temperature rhythm (treatment × time: F _[1, 7]_ = 8.76, *p* = .02). On the other hand, the time-of-day-dependent effect was not observed in DTA narcoleptic mice (treatment × time: F _[1, 7]_ = 0.64, *p* = .45; Figure 1C).

#### Locomotor activity.

In previous studies, TA supplementation dose-dependently increased locomotor activity during the dark period, especially the second half of the dark period [12, 13]. Similarly, there was an increase in locomotor activity by TA supplementation during the second half of the dark period in WT mice (control vs 0.5% TA at ZT0-5: 2334.5 ± 663.8 vs 2161.8 ± 356.0, *p* = .99; ZT6-11: 1830.3 ± 186.0 vs 1821.9 ± 164.6, *p*  ≥ .99; ZT12-17: 6026.1 ± 606.9 vs 6833.8 ± 1105.3, *p* = .60; ZT18-23: 5618.5 ± 763.9 vs 7449.6 ± 983.9, *p* = .03; Figure 1D). Compared to WT mice, DTA mice showed reduced overall activity during the dark period, resulting in dampened diurnal activity rhythms (Figure 1E). TA supplementation induced a mild increase in locomotor activity at the onset of the dark period, although no statistically significant differences between treatments were observed (control vs 0.5% TA at ZT0-5: 3049.9 ± 358.8 vs 2506.4 ± 379.2, *p* = .53; ZT6-11: 2738.9 ± 335.5 vs 2280.8 ± 224.6, *p* = .53; ZT12-17: 4284.6 ± 477.1 vs 5373.3 ± 631.0, *p* = .24; ZT18-23: 4415.4 ± 546.2 vs 4114.4 ± 820.0, *p* = .98).

### TA supplementation consolidated wakefulness during the active period

**Figure 2.**
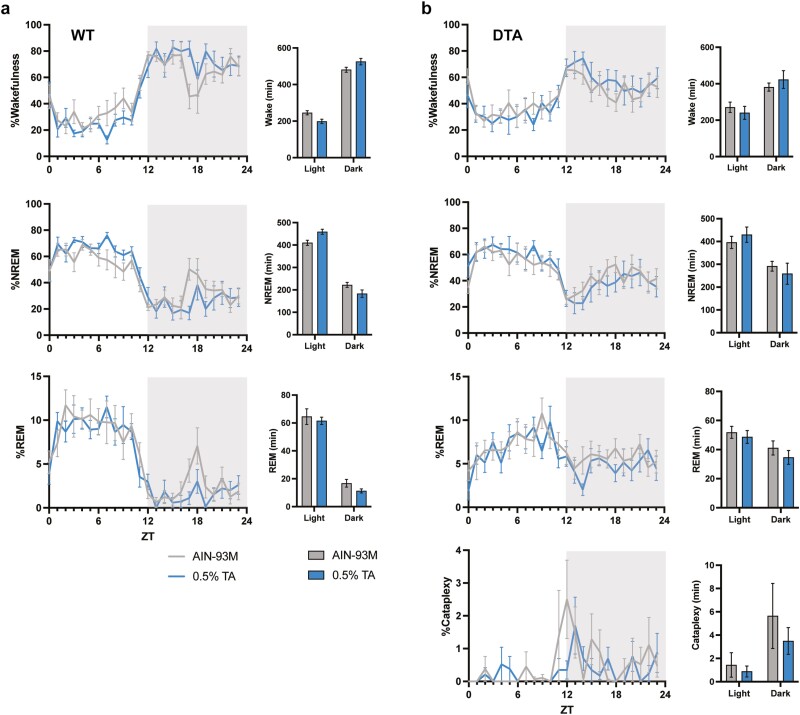
Behavioral states in a 24-hour period. Percentage time spent in wake, NREM, REM, and DREM across light–dark cycle in (A) WT and (B) DTA mice. The gray and blue lines represent the group fed with control AIN-93M diet or 0.5% TA-supplemented diet, respectively. One WT mouse was excluded from scoring due to the EEG noises/artifacts. All data are presented as mean ± SEM (*n* = 7 for WT mice and *n* = 8 for DTA mice). Data were compared between the treatments over 24 hours (in 12-hour bins) by two-way repeated measures ANOVA. *p < .05.

**Figure. 3.**
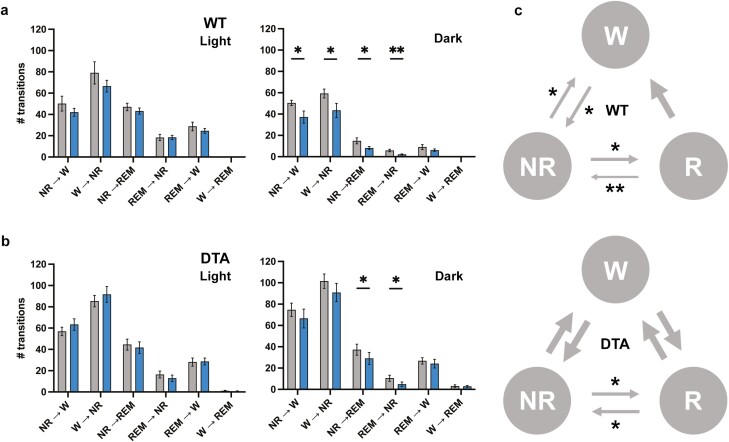
Sleep and wake transitions. The number of transitions between behavioral states in the light and dark periods were analyzed in WT (A) and DTA (B) mice. (C) Diagram for wakefulness (W)-NREM sleep (NR)-REM sleep (R) transitions during the dark period compared to the control diet. The diagram during the light period was omitted because no statistical difference was observed in both WT and DTA mice. The thickness of the arrows is proportional to the corresponding statistical significance. All data are presented as mean ± SEM (*n* = 7 for WT mice and *n* = 8 for DTA mice). *p < .05, **p < .01 significant difference relative to the control diet.

Similar to previous studies, TA supplementation showed a tendency to increase wakefulness during the dark period and had a time-of-day-dependent effect on wakefulness and NREM sleep (treatment × time: wake, *p* = .02; NREM, *p* = .01; REM, *p* = .70; Figure 2A, Table 1). While TA induced the time-of-day-dependent change in the wake and NREM sleep amount between the light and dark periods, the difference did not reach a criteria for statistical significance (wake time at light, control vs 0.5% TA: 245.8 ± 11.3 minutes vs 199.0 ± 11.7 minutes, *p* = .07; wake at dark: 481.2 ± 13.8 minutes vs 525.6 ± 17.7 minutes, *p* = .10; NREM at light: 409.7 ± 11.4 minutes vs 459.5 ± 11.3 minutes, *p* = .05; NREM at dark: 222.0 ± 11.2 minutes vs 183.0 ± 16.7 minutes, *p* = .13). However, the average duration of wake and NREM was significantly longer with TA supplementation during the dark and light periods, respectively, suggesting that TA supplementation can promote the maintenance of long bouts of wake and NREM during each period (Table 2). On the other hand, DTA mice showed attenuated responses to TA supplementation in terms of the amount of change in each behavioral state, resulting in the disappearance of the time-of-day-dependent effect (treatment × time: wake, *p* = .09; NREM, *p* = 0.08; REM, *p* = .58; Figure 2B, Table 1). TA supplementation did not effectively suppress the amount and bout number of cataplexy. Furthermore, the impaired ability of DTA mice to sustain long bouts of wake during the dark period was not sufficiently improved by TA supplementation, despite a tendency toward long wake bouts during the active period (Table 2). We also examined transitions between behavioral states to further compare sleep–wake architecture between control and TA extract-supplemented diet. TA supplementation influenced transitions during the dark period, but not the light period, in both WT and DTA mice (Figure 3, A and B). As evidenced by the small number of wake bouts in WT mice in Table 2, the transitions from wake to NREM and NREM to wake significantly decreased in TA supplementation (W to NR: 59.3 ± 4.1 vs 43.4 ± 6.7, *p* = .01; NR to W: 50.4 ± 2.5 vs 37.1 ± 5.7, *p* = .03). The transitions from NREM to REM and REM to NREM also significantly decreased in TA supplementation (NR to R: 14.9 ± 2.8 vs 8.1 ± 1.4, *p* = .02; R to NR: 5.9 ± 1.2 vs 2.1 ± 0.6, *p* = .003), in part because of the lower chance of NREM bouts. In DTA mice, the frequent transitions from wake to NREM and NREM to wake during the active period were not improved by TA supplementation (W to NR: 101.5 ± 6.8 vs 90.9 ± 8.6, *p* = .37; NR to W: 74.6 ± 6.3 vs 66.5 ± 8.9, *p* = .44). The transitions from NREM to REM and REM to NREM decreased in DTA mice (NR to R: 37.3 ± 5.2 vs 29.0 ± 5.6, *p* = .03; R to NR: 10.5 ± 2.8 vs 4.9 ± 2.0, *p* = .02). The state transitions in the dark period are summarized in Figure 3C.

**Table 1.** Treatment × Time Interaction in WT and DTA Mice

	WAKE	NREM	REM	CATAPLEXY
WT	F (1,6) = 10.1	F (1,6) = 12.1	F (1,6) = 0.16	
	*p* = .02	*p* = .01	*p* = .70	
DTA	F (1,7) = 3.85	F (1,7) = 4.03	F (1,7) = 0.34	F (1,7) = 1.60
	*p* = .09	*p* = .08	*p* = .58	*p* = .25

**Table 2.** The Number and Mean Duration of Each Vigilance State

		WAKE		NREM		REM		CATAPLEXY	
		Light	Dark	Light	Dark	Light	Dark	Light	Dark
*Number of bouts*									
WT	AIN-93M	79.6 ± 10.5	60.0 ± 4.1	97.6 ± 8.9	65.6 ± 4.4	47.0 ± 3.5	14.9 ± 2.8		
	0.5% TA	67.0 ± 5.6	43.7 ± 6.6*	85.4 ± 5.8	45.9 ± 6.5**	43.1 ± 2.9	8.3 ± 1.3*		
DTA	AIN-93M	86.9 ± 5.6	105.0 ± 6.7	101.9 ± 5.5	112.5 ± 7.7	44.5 ± 5.1	37.3 ± 5.2	1.0 ± 0.6	3.1 ± 1.2
	0.5% TA	92.9 ± 7.3	94.4 ± 8.6	105.3 ± 7.2	96.0 ± 8.2	41.5 ± 5.4	29.0 ± 5.6	0.6 ± 0.4	3.1 ± 0.8
*Bout duration*									
WT	AIN-93M	209.1 ± 33.4	497.3 ± 43.1	260.4 ± 16.5	205.1 ± 10.3	83.3 ± 7.4	69.0 ± 5.6		
	0.5% TA	190.4 ± 30.5	820.3 ± 119.1*	331.3 ± 23.5*	253.7 ± 28.6	86.0 ± 3.1	84.0 ± 4.2		
DTA	AIN-93M	191.6 ± 21.5	224.8 ± 22.4	235.8 ± 18.9	158.9 ± 14.9	72.6 ± 4.7	68.6 ± 6.0	69.0 ± 29.6	100.1 ± 27.7
	0.5% TA	162.1 ± 27.2	298.3 ± 56.7	247.9 ± 20.7	155.8 ± 21.2	75.5 ± 7.5	78.1 ± 7.1	93.3 ± 12.0	63.4 ± 12.8

**Figure 4.**
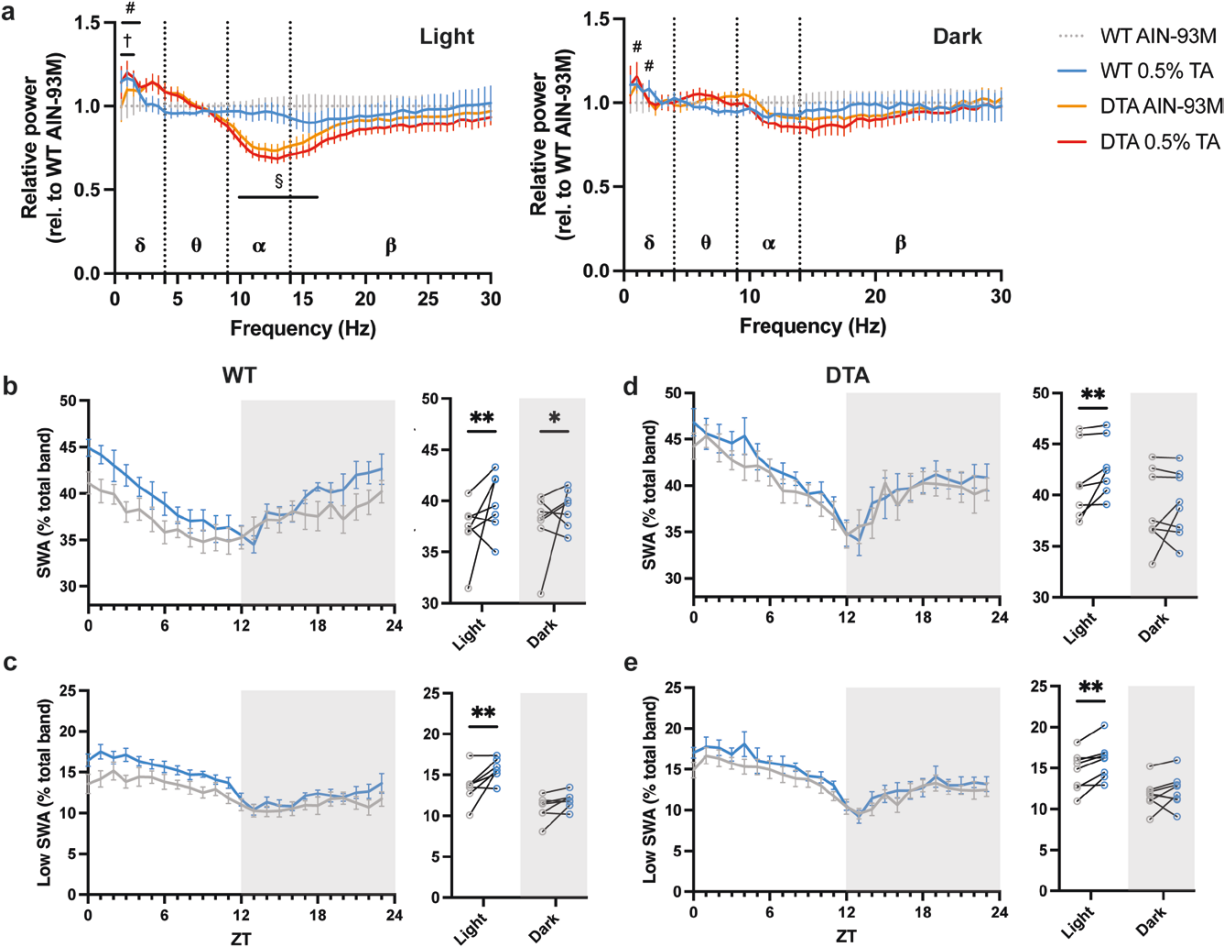
FFT power spectral analysis during NREM sleep. (A) Comparison of the relative spectral power during NREM sleep among groups in light and dark periods. NREM EEG profiles were analyzed across multiple frequency ranges (δ [0.5–4 Hz], θ [4–9 Hz], α [9–14 Hz], and β [14–30 Hz]). Time course of percentages of SWA at delta (0.5–4.0 Hz) and low delta (0.5–2.0 Hz) frequencies across 24 hours in (B, C) WT and (D, E) DTA mice. All data are presented as mean ± SEM (*n* = 7 for WT mice and *n* = 8 for DTA mice). #*p* < .05 compared between the AIN-93M and 0.5% TA treatment in WT mice; ^†^*p* < .05 compared between the AIN-93M and 0.5% TA treatment in DTA mice; ^§^*p* < .05 compared between the AIN-93M treatment of each genotype; *p < .05, **p < .01 compared to the relevant control diet.

Next, we performed FFT analysis to examine the EEG spectral profiles during NREM sleep. WT mice showed increased slow wave activity (SWA), power in the delta frequency range (0.5–4.0 Hz), during both the light and dark periods under TA supplementation (Figure 4A). In particular, TA extract significantly enhanced power at lower frequencies between 0.5 and 2.0 Hz during the light period, accounting for a considerable amount of the increase of SWA (control vs 0.5% TA for SWA: light, 37.2% ± 1.1% vs 39.8% ± 1.1%, *p* = .005; dark, 37.7% ± 1.2% vs 39.3% ± 0.7%, *p* = .04; for low SWA: light, 13.6% ± 0.8% vs 15.7% ± 0.5%, *p* = .001; dark, 10.9% ± 0.6% vs 11.9% ± 0.4%, *p* = .05; Figure 4, B and C). At baseline, delta and alpha frequencies were significantly changed in DTA mice compared to WT mice (Figure 4A). Interestingly, DTA mice also showed an increase in SWA by TA supplementation during the light period (Figure 4, A and D). Likewise, the increased SWA was mostly explained by the increase in lower frequencies of SWA in DTA mice (control vs 0.5% TA for SWA: light, 41.2% ± 1.2% vs 42.5% ± 0.9%, *p* = 0.003; dark, 38.6% ± 1.3% vs 39.1% ± 1.1%, *p* = .16; low SWA: light, 14.7% ± 0.8% vs 15.9% ± 0.8%, *p* = .004; dark, 11.8% ± 0.6% vs 12.3% ± 0.7%, *p* = .20; Figure 4E). SWA at lower frequencies is known to be sensitive to changes in arousal and exploratory behavior during the proceeding active period [21, 22]. Indeed, TA enhanced locomotion during the dark period in WT mice (Figure 1D).

### TA increased a corticosterone/testosterone ratio

#### Testosterone.

**Figure 5.**
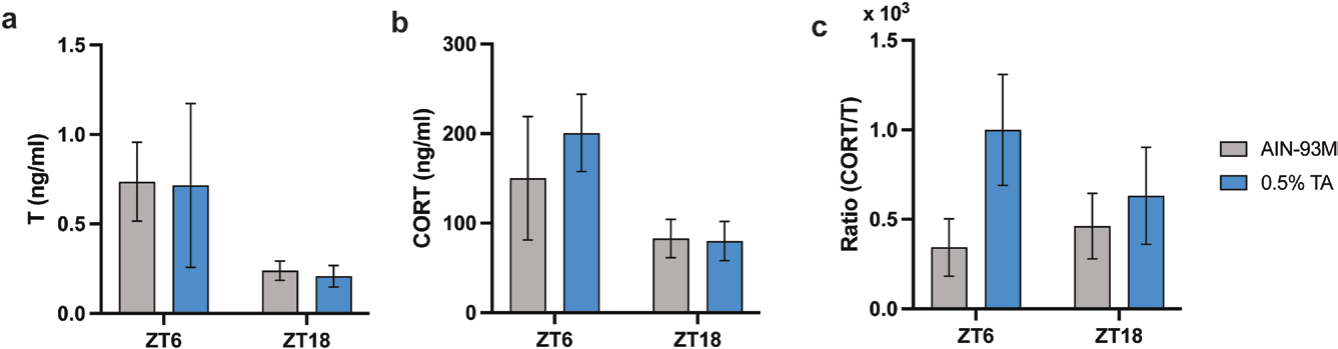
Serum testosterone (T) and corticosterone (CORT) levels. (A) Testosterone and (B) corticosterone were analyzed in serum samples collected at ZT 6 and ZT 18. (C) The ratio was calculated accordingly. All data are presented as mean ± SEM (*n* = 5 for each group).

Serum hormone level in C57BL/6 mice was measured using ELISA. Unlike clinical studies, TA did not increase the testosterone level at ZT 6 nor ZT 18 (Figure 5A).

#### Corticosterone.

TA showed an increasing tendency of the corticosterone level at ZT 6, but not at ZT 18 (Figure 5B). Since the restored hormone balance was reported in clinical studies [2, 5], a ratio of corticosterone to testosterone was calculated. The ratio increased in the TA-supplemented group at ZT 6, although there was no statistical difference due to high variability (ZT = 6, *p* = .40; ZT = 18, *p* = .69; Figure 5C).

### Hypocretin neuron was not activated by TA

**Figure 6.**
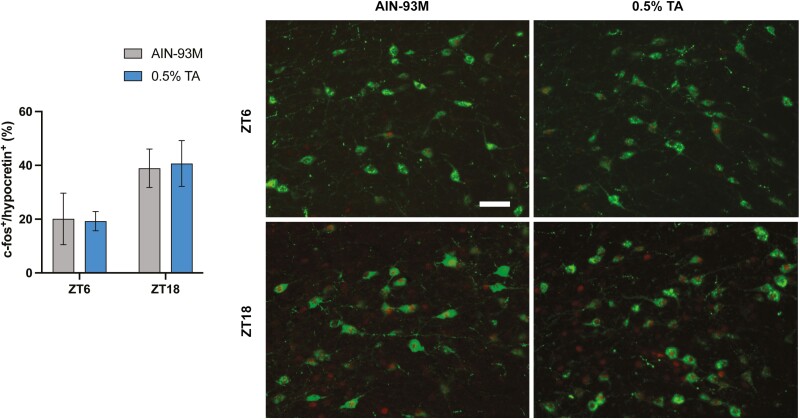
c-FOS immunostaining in hypothalamic hypocretin neurons. A series of coronal sections, including the lateral hypothalamus, were stained with anti-hypocretin and c-fos antibodies. Percentage of c-fos^+^ and hypocretin^+^ cells out of total hypocretin^+^ cells were shown. All data are presented as mean ± SEM (*n* = 5 for each group). Representative images showing c-Fos expression (red nuclei) within hypocretin neurons (green soma). Scale bar = 100 µm.

The poor responsiveness to TA treatment in DTA mice led us to test whether hypocretin neurons can be activated by TA supplementation in C57BL/6 mice. To evaluate the activated hypocretin neurons in the lateral hypothalamus, c-fos^+^/hypocretin^+^ double-positive cells were counted. There were no statistical differences observed between the control and TA groups in ZT 6 nor ZT 18 (Figure 6).

## Discussion

We confirmed and extended previous studies examining time-of-day-dependent effects of TA extract supplementation on temperature, locomotion, and sleep–wakefulness in WT and narcoleptic DTA mice [12, 13]. We found that dietary TA supplementation intensified diurnal rhythms of locomotion and temperature in a time-of-day-dependent manner in WT mice but attenuated in DTA mice. The consolidating effect on behavioral states did not sufficiently improve the disturbed sleep and wake cycle nor cataplexy in DTA mice. These results suggest that TA is not as potent as wake-promoting drugs to improve pathological sleepiness in murine narcolepsy. In general, attenuated wake-promoting effects of psychostimulants, such as caffeine and modafinil, are observed in mouse models of narcolepsy [20, 23]. Caffeine is widely used to improve alertness by not only healthy people but also patients with hypersomnia before the diagnosis is made. However, its potency is not strong enough for excessive daytime sleepiness in narcolepsy. Interestingly, our results suggested that the increase in SWA during the resting period may be indicative of a homeostatic response to increased activity and vigilance during the active period. In this regard, TA supplementation still offers potential benefits for improving daily rest–activity rhythm in healthy participants.

Interestingly, it has been reported that chronic caffeine treatment also causes a similar increase in daily sleep–wake cycle amplitude in mice. Chronic caffeine abolishes the siesta and consolidates wakefulness during the active period, and the mice sleep more solidly during the resting period [24]. In addition, chronic caffeine intake can increase sleep pressure, evidenced by difficulties in keeping mice awake during sleep deprivation [25]. Although we did not perform sleep deprivation under chronic TA treatment, the notion that sleep pressure is increased by chronic TA during the light period is supported by the finding in WT mice that NREM sleep duration was also increased during this period. In contrast, the increase in SWA is more likely complicated in DTA mice due to the absence of a corresponding increase in the duration of wakefulness. This raises the possibility that TA administration itself deepens sleep intensity along with homeostatic responses. Considering the similarity in the long-term effects to caffeine, TA might also influence sleep amount and need by modulating the adenosine system. However, DTA mice have shown altered homeostatic sleep responses, compared to WT mice, to wake-promoting compounds during the dark period [20], and thus these findings warrant further investigation.

TA extract has traditionally been utilized as a general health tonic for anti-aging and aphrodisiac benefits. It contains a wide range of bioactive compounds including quassinoids and alkaloids [26]. While the mechanism of action remains largely unknown, restoration of hormone balance (cortisol/testosterone) has been proposed to be involved in these effects in moderately stressed participants [2]. A clinical study claims that TA extract increases testosterone levels in women not only by stimulating testosterone biosynthesis, but also by the decline in serum sex-hormone-binding-globulin concentrations, contributing to an increased release rate of free testosterone [27]. However, there was no increase in the testosterone level with TA supplementation in this study. One study using male rats aged 12 months also failed to increase testosterone after 6 weeks of the treatment [28]. Another study using young rats reported elevated testosterone levels accompanied by elevated aphrodisiac activities [29]. The discrepancy in testosterone levels between human and rodent studies may be explained, in part, by variations in the basal stress level in participants; elderly participants with certain levels of perceived stress were recruited in the clinical studies [2, 5, 27]. Indeed, testosterone levels at the age of 60 are typically only half of the youthful levels, at the peak between 25 and 30 years of age, and they may be lower due to stress and related lifestyle issues such as diet, exercise, and sleep patterns [30–33]. Although there was no significant change observed in testosterone and corticosterone levels, the ratio of corticosterone to testosterone tended to be higher in the TA supplementation group than in the control group, especially during the resting period. In general, hormone imbalance is associated with alterations in sleep patterns and vice versa [34]. We recently reported that TA did not aid or interfere with transitioning back to sleep after acute sleep disturbance, as assessed by exchanging cages during the resting period, suggesting that chronic TA treatment is not likely a long-term stressor [13]. Considering the negative correlation between corticosterone and testosterone, it is still not clear how hormone balance restoration can be involved in the beneficial effect of TA extract in clinical studies.

The activation of hypocretin neurons induces wakefulness, while their loss is associated with narcolepsy. The poor responsiveness to TA treatment in DTA mice led us to evaluate whether hypocretin neurons can be a target of TA to consolidate behavioral states. However, we found that hypocretin neurons were equally activated between treatments in a time-of-day dependent manner. Therefore, it appears TA may target other neuronal populations to modulate sleep and wakefulness. The notable effect of TA on core body temperature raises interest in exploring the possible involvement of neuronal populations in the preoptic area, known to regulate sleep-induced hypothermia, as potential targets for TA action [35, 36]. One study reported that cortical and hippocampal dopamine levels dose-dependently increased, but not other monoamines, in the rat brain after TA extract administration [29]. The elevated dopamine level may reflect an increase in motivation and arousal. Our recent study in mice has also reported that TA extracts slightly lengthen the circadian period in behavioral rhythms, and may act on circadian clock mechanisms, which may lead to increased rest–activity amplitude [13]. Since its effects on brain functions are still largely unexplored, these studies warrant further investigation to examine the mechanism of how TA extract can modify behavioral states.

The study’s main limitation is that there are some inconsistencies with prior studies in terms of the potency of TA on the rest–activity rhythm enhancement [12, 13]. One likely possibility is a variability of the actual dose taken by mice throughout the chronic treatment. All studies used a similar protocol to prepare TA-containing pellets. Unfortunately, we did not measure their body weight and food consumption in this study, and we cannot exclude this possibility. In fact, eurycomanone, a bioactive quassinoid, is poorly bioavailable when given orally [37]. This poor oral bioavailability of the potential functional ingredient may affect tissue concentration, which can result in variability. Another possibility is the age differences used for experiments. Miyazaki et al. used 2-month-old mice, while the study by Ono et al. and the current study used middle-aged mice at 8 months and 10–12 months old, respectively. The influence of genetic background and food manipulation during the developing period also need to be considered. The current study used WT littermates of DTA mice with mixed C57BL/6 background [20], while the others used pure C57BL/6 strains. WT mice were fed with doxycycline-containing chow until 7 weeks old. Although it is unknown if doxycycline treatment during the development affects food consumption and preference in adults, these differences in experimental conditions, regarding the compound and mice, may explain the inconsistencies in potency between studies. Another important limitation is that the FOS experiment was performed under a chronic condition. Due to the nature of the FOS expression pattern, a bolus of TA would be straightforward to examine the activation of wake-promoting neurons. Since an effective dose of bolus injection has not been determined, we performed the FOS experiment under chronic TA treatment to compare the basal expression level.

In summary, TA extract exhibits a unique time-of-day-dependent effect on wake and sleep patterns, although it was not as potent as wake-promoting drugs to improve pathological sleepiness in murine narcolepsy. The causal relationship between the time-of-day-dependent effect and the altered hormone balance remains inconclusive. However, its pharmacological properties, with few side effects, would be potentially beneficial as a tonic, especially for middle-aged and elderly individuals, who commonly experience destabilization of wakefulness and NREM sleep, along with increased light sleep during the resting period, similar to sleep disturbances observed in aged rodents [14–16]. Thus, TA extract may have significant potential for restoring and enhancing the sleep/wake rhythm in the general adult population. To date, there have been only a very limited number of clinical trials evaluating the effects of TA supplementation on daytime sleepiness or nocturnal sleep. Therefore, further clinical trials and mechanistic studies are warranted to determine its potential benefits on sleep and alertness.

## Data Availability

The data that support the findings of this study are available from the corresponding author upon reasonable request.
